# Fluorine-Substituted Arylphosphine for an NHC-Ni(I) System, Air-Stable in a Solid State but Catalytically Active in Solution

**DOI:** 10.3390/molecules24183222

**Published:** 2019-09-04

**Authors:** Kouki Matsubara, Takahiro Fujii, Rion Hosokawa, Takahiro Inatomi, Yuji Yamada, Yuji Koga

**Affiliations:** Department of Chemistry, Fukuoka University, 8-19-1 Nanakuma, Fukuoka 814-0180, Japan (T.F.) (R.H.) (T.I.) (Y.Y.) (Y.K.)

**Keywords:** monovalent nickel, fluorine-substituted phosphine, Kumada coupling, intermolecular interaction, DFT calculations

## Abstract

Monovalent NHC-nickel complexes bearing triarylphosphine, in which fluorine is incorporated onto the aryl groups, have been synthesized. Tris(3,5-di(trifluoromethyl)-phenyl)phosphine efficiently gave a monovalent nickel bromide complex, whose structure was determined by X-ray diffraction analysis for the first time. In the solid state, the Ni(I) complex was less susceptible to oxidation in air than the triphenylphosphine complex, indicating greatly improved solid-state stability. In contrast, the Ni(I) complex in solution can easily liberate the phosphine, high catalytic activity toward the Kumada–Tamao–Corriu coupling of aryl bromides.

## 1. Introduction

Recent developments in the field of nickel catalysis have opened up possibilities of new catalytic processes directly involving monovalent nickel complexes in organometallic chemistry [1,2,3,4,5,6] along with those involving conventional zerovalent nickel catalysts. Although limited to the structures of specific ligands and complexes, monovalent nickel complexes are thermally stable and isolable [7]. Several studies have used well-defined nickel(I) complexes as catalyst precursors [8,9,10,11,12,13,14], and reactions where nickel(I) complexes are the key intermediates have been proposed based on theoretical calculations [15,16,17,18,19,20]. Recently, experimental results have also supported the catalyst chemistry of nickel(I) [21,22,23]. One of the current bottlenecks in nickel(I) catalysis research is developing catalytically active, air-stable nickel(I) catalyst precursors. It is safe to say that a breakthrough here would go a long way in furthering our knowledge about nickel catalyst chemistry.

To date, phosphines with fluorine-substituted aryl groups have been frequently used due to their attractive features in many catalytic reactions [24,25,26,27,28,29,30,31]. The induced electron-withdrawing property of fluorine increases the π-acceptor nature of the phosphorus ligand, and it is expected that the back-donation from the metal stabilizes the electron-rich, low-valent metal center. Cone-angles relatively larger than those of phosphite ligands also provide kinetic stabilization. It may be noted that tris(3,5-di(trifluoromethyl)phenyl)phosphine (P(Ar*_m_*-_CF3_)_3_) has additional unique properties in metal complexes [24,25,26,27,28]. In this case, fluorine atoms can induce unique CF···π interactions between CF_3_ groups and aromatic rings of ligand molecules to form solid ligand structures that stabilize unsaturated metal complexes such as the “superstable” Pd(0) catalyst, [Pd(P(Ar*_m_*-_CF3_)_3_)_3_] [29].

**Scheme 1:**
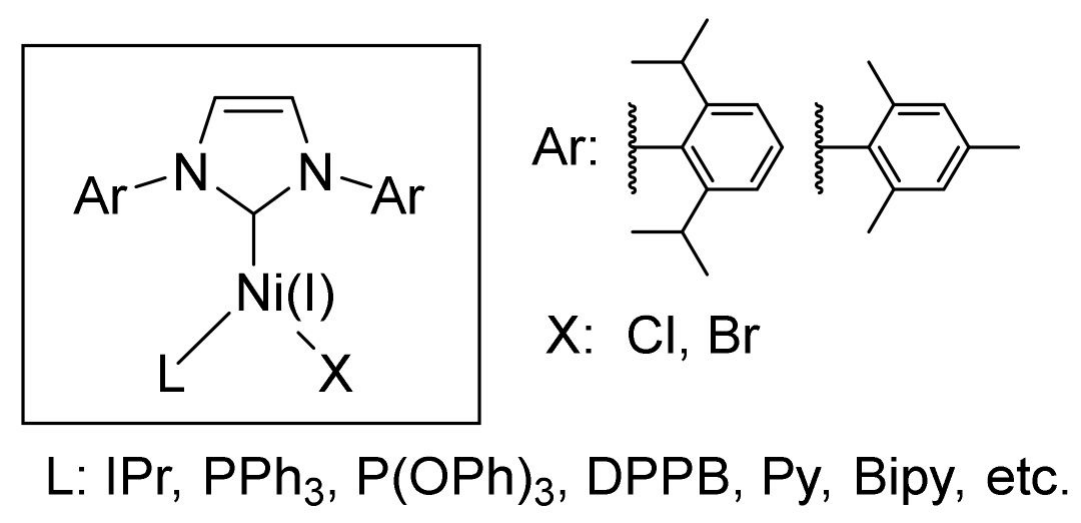
Previously reported mononuclear Ni(I)-NHC halide complexes [8].

Recently we have synthesized three-coordinated mononuclear nickel(I) complexes [Ni(IPr)Cl(L)] (IPr = 1,3-bis(2,6-diisopropylphenyl)imidazol-2-ylidene) by adding various monodentate or bidentate ligands L to the dinuclear nickel(I) complex, [Ni(IPr)Cl]_2_ [32,33,34]. The series of complexes shown in Scheme 1 works efficiently under mild conditions in cross-coupling reactions, such as Buchwald–Hartwig amination, Kumada–Tamao–Corriu coupling, and Suzuki–Miyaura coupling [33,34]. These mononuclear complexes can regenerate the dinickel(I) halide complex in solution, which can generate coordinatively unsaturated active intermediate, mononuclear Ni(I) NHC complex, [Ni(NHC)X] [21]. When using weakly coordinating pyridine as the stabilizing ligand, the stability of Ni(I) proved to be insufficient, whereas strongly withdrawing phosphite, P(OPh)_3_, lowered its catalytic performance [34]. The complexes containing usual phosphines like PPh_3_ were also smoothly oxidized even in the solid state. Therefore, to construct more stable and active nickel(I) complexes, investigation of alternative ligands, such as the *m*-CF_3_-substituted arylphosphine (P(Ar*_m_*-_CF3_)_3_), is necessary. However, to the best of our knowledge, there has been no report on well-defined nickel complexes bearing this phosphine ligand.

In the course of our research, we have found a desirable ligand (fluorine-substituted monodentate phosphine) to stabilize three-coordinated nickel(I) complex. Intriguingly, when using P(Ar*_m_*-_CF3_)_3_, the obtained monovalent nickel complex in the crystalline state did not suffer air oxidation for some minutes, while simultaneously maintaining the catalytic activity in the Kumada–Tamao–Corriu cross-coupling reaction in solution under an inert-gas atmosphere. Here, we have reported the synthetic procedure, structures, and properties of the nickel(I) complexes and results in a Kumada–Tamao–Corriu cross-coupling reaction. This is the first example of a well-defined nickel complex bearing the *m*-CF_3_-substituted triarylphosphine.

## 2. Results

### 2.1. Preparation and Characterization of Ni(I) Complexes

**Scheme 2:**
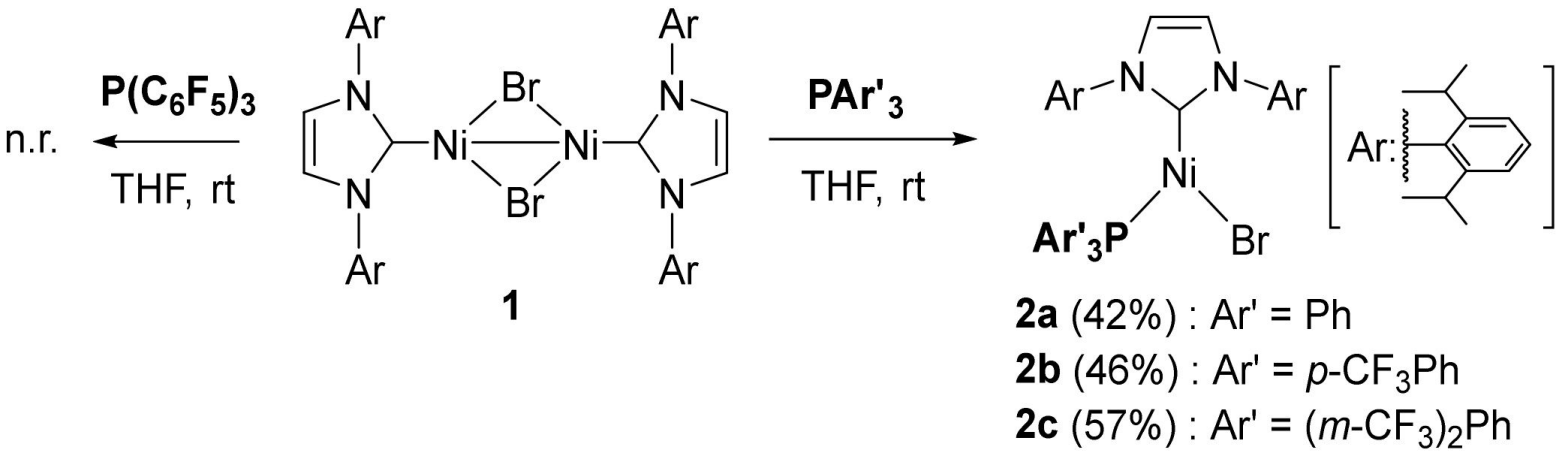
Reaction of dinickel(I) complex **1** with phosphines.

The bromide analogue of [Ni(IPr)Cl]_2_ reported in the literature [35] is expected to be more stable than its chloride analogue and can be transformed into mononuclear nickel(I)-IPr complex in conjunction with the phosphorus ligand. The reactions of [Ni(IPr)Br]_2_ (**1**) with triarylphosphines, triphenylphosphine (PPh_3_), tris(4-(trifluoromethyl)phenyl)phosphine (P(Ar*_p_*-_CF3_)_3_), tris(3,5-bis(trifluoromethyl)phenyl)phosphine (P(Ar*_m_*-_CF3_)_3_), and tris(pentafluorophenyl)phosphine (P(C_6_F_5_)_3_) were conducted in a tetrahydrofuran (THF) solution at room temperature. As expected, the corresponding mononuclear nickel(I) complexes, [NiBr(IPr)(PAr_3_)] (PAr_3_ = PPh_3_ (**2a**), P(Ar_p-CF3_)_3_ (**2b**), and P(Ar*_m_*-_CF3_)_3_ (**2c**)), were successfully obtained in 42, 46, and 57% yields (Scheme 2), respectively, after recrystallization. However, P(C_6_F_5_)_3_ did not coordinate to nickel, resulting in the complete recovery of the starting materials under the reaction conditions. This may be attributed to steric repulsion between a pair of diisopropylphenyl groups of the IPr ligand and pentafluorophenyl groups of P(C_6_F_5_)_3_, where the cone angle (184°) is greater than that of P(Ar*_m_*_-CF3_)_3_ (160°).

The obtained Ni(I) complexes were paramagnetic and the EPR resonances were observed even at room temperature (see Supporting Information), similar to the previous chloride analogue, [NiCl(IPr)(PPh_3_)] [33]. Moreover, a SQUID (superconducting quantum interference device) measurement of **2c** showed a favorable value of *χ*_M_*T* = 0.47 cm^3^·K·mol^−1^ at 50 K, assigned as S = 1/2. The value did not change over the temperature range until 300 K, according to Curie′s rule. The previous study also showed similar values (*χ*_M_*T* = 0.35–0.52 cm^3^·K·mol^−1^), which are somewhat higher than the theoretical value of 0.375 cm^3^·K·mol^−1^ when S = 1/2, attributed to spin-orbital angular momentum.

The ^1^H-NMR spectral measurement for the crystals of **2a**–**2c** dissolved in C_6_D_6_ revealed a partial regeneration of the starting complex **1** accompanied by free triarylphosphine from these products. A similar phenomenon is observed in a solution of the previous Ni(I) chloride analogues, suggesting the existence of coordination-elimination equilibrium of weakly coordinating triarylphosphine in solution [34]. It is notable that the ratio of the regenerated complex **1** from the mononuclear complex **2c** was only slightly smaller than the ratio from **2a**.

### 2.2. X-ray Crystallography and Theoretical Studies

**Figure 1:**
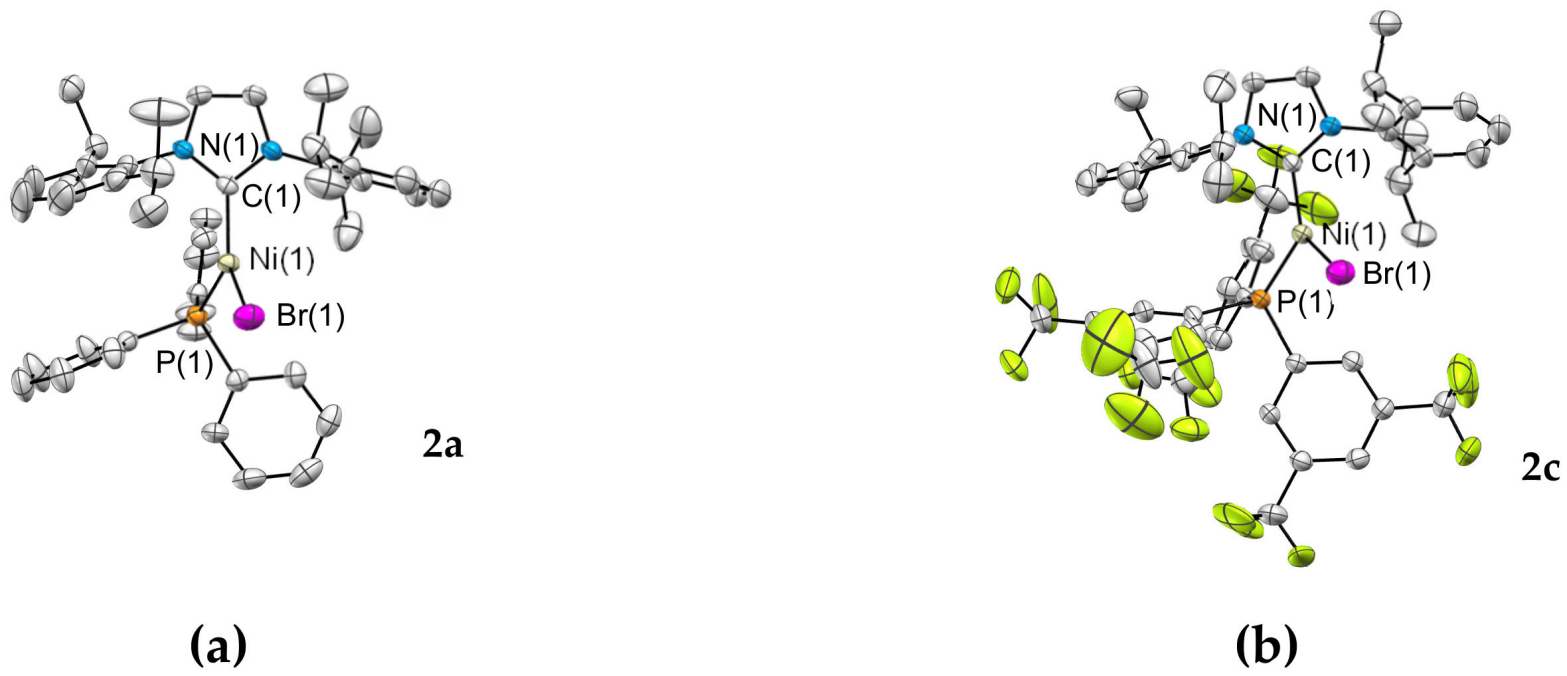

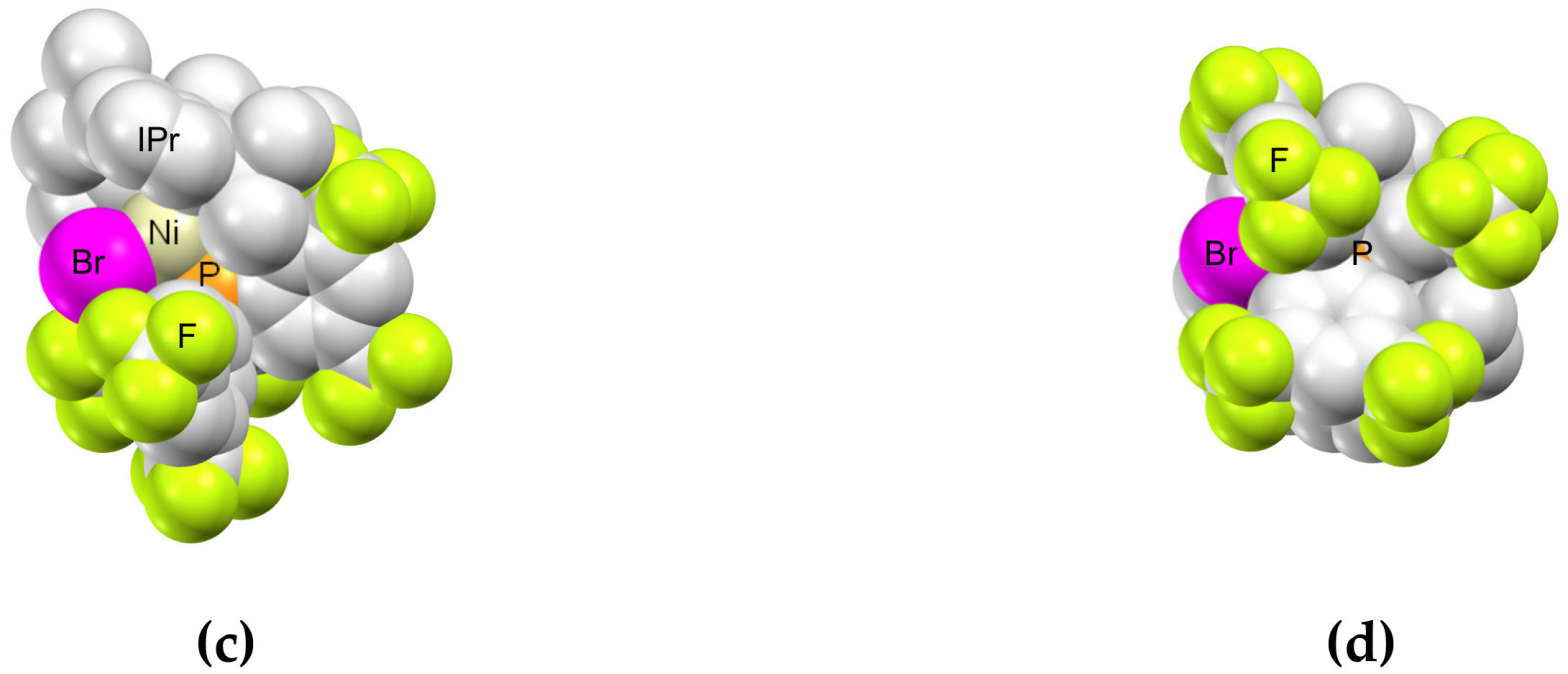
ORTEP drawing of (**a**) **2a** and (**b**) **2c** (50% probability of thermal ellipsoids. Hydrogen atoms and solvent molecule (THF in the crystal of **2c**) are omitted for clarity). Views of space-filling model of **2c** from (**c**) the side and (**d**) the bottom are illustrated (pink: Br; gray: C; light green: F; orange: P; light yellow: Ni).

The structures of complexes **2a**, **2b**, and **2c** were confirmed by X-ray crystallography by using single crystals after recrystallization. Figure 1 shows representative examples of the structures of **2a** and **2c**. The result from the crystal of **2b** was insufficient to discuss the detailed structure, unfortunately. There were no significant differences between the **2a** and **2c** structures, as far as the bond lengths and angles of nickel–bromine bonds are concerned. (Table 1). On the other hand, some differences were observed in the bond lengths between the nickel–phosphorus and the nickel–carbene carbon atoms: Ni(1)–P(1), 2.201(1) (**2a**) and 2.182(1) (**2c**); Ni(1)–C(1), 1.918(5) (**2a**), and 1.936(3) Å (**2c**). Similar differences between the metal–phosphorus atom in platinum(II) complexes bearing PPh_3_ and P(Ar*_m_*-_CF3_)_3_ were reported as ca. 0.02 Å [36]. The extension of the nickel–carbon bond in **2c** appears to be in balance with the strength of the back-donation to the nickel–phosphorus bond.

A space-filling model of **2c** was depicted in Figure 1c,d to show the coordination sphere of nickel. The substituents of P(Ar*_m_*-_CF3_)_3_ largely occupied the remaining spaces other than IPr and bromine around nickel. In particular, one CF_3_ on the aryl group is sandwiched between the two isopropyl groups contained in the two wingtip groups of IPr, which hampers the free rotation of the Ni–P axis. If so, the ^19^F signals from the CF_3_ groups could not be equivalently observed. The averaged equivalent ^19^F signal of **2c** may be provided by the fast equilibrium of elimination and the coordination of phosphine. Because this complex has only one phosphine ligand, intramolecular CF···π interaction between phosphine ligands did not exist in the crystal structure.

**Figure 2:**
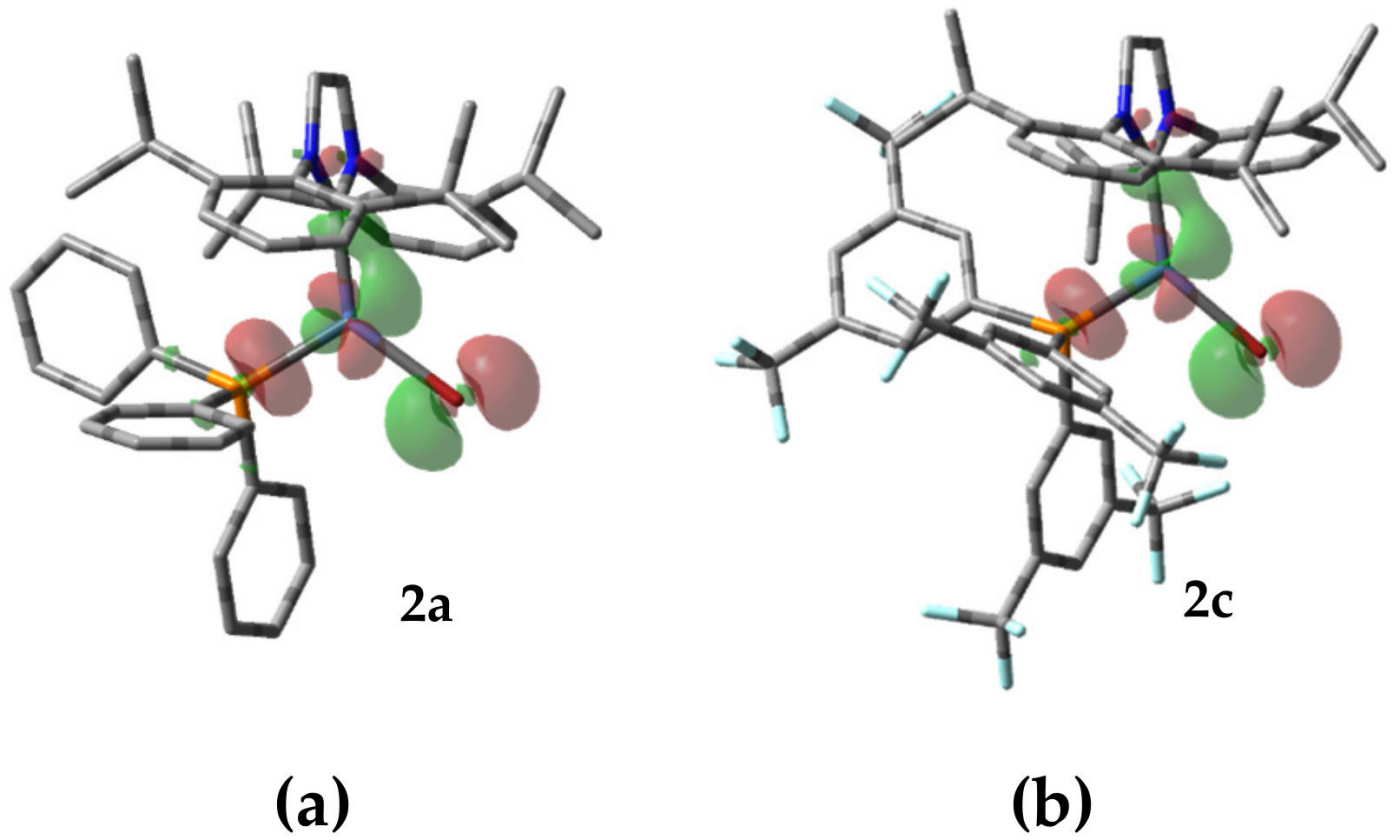
Kohn–Sham orbitals of SOMO corresponding to a density isosurface value of 0.02 au in (**a**) **2a** and (**b**) **2c** obtained from single-point DFT calculation with B3LYP/6-31G(d,p) level using the crystallographic coordinates.

The distribution of the single electron-occupied molecular orbital (SOMO) of **2a** and **2c** was investigated by performing single-point density functional theory (DFT) calculations (B3LYP/6-31G(d,p) level) implemented in the Gaussian 16 program package [37] at the fixed geometries given by the crystallographic coordinates. Similar electron distributions were given in both complexes as shown in Figure 2a,b. The unpaired electron in SOMO is distributed mainly in one d orbital of nickel, resulting in the formation of two σ*(d-n_σ_) orbitals with σ-type non-bonding orbitals (n_σ_) of the phosphorus atom and the NHC unit, and the π*(d-p) orbital between nickel and bromine. Because of the electron-withdrawing property of the CF_3_ groups, the acceptor ability of the arylphosphine may be enhanced in **2c**. The ratio of the unpaired electron in the nickel d-orbital was 34.9% in **2c**, lower than that of **2a** (41.4%), whereas that in the phosphorus atom was 12.4% and 10.6% in **2c** and **2a**, respectively. Interestingly, in the case of bromine, the figure was much larger in **2c** (28.3%) than that in **2a** (21.7%). The reason for this may be the fact that the energy level of NHC–Ni–PPh_3_ unit in **2c** providing the main component of SOMO is lower-shifted upon the substitution of the CF_3_ group into arylphosphine to become closer to the 4p orbital of bromine. This can lead to a greater occupation of the 4p orbital in the Ni–Br π*-orbital component of SOMO as compared to that of **2a**.

### 2.3. Oxidation Potentials

**Figure 3:**
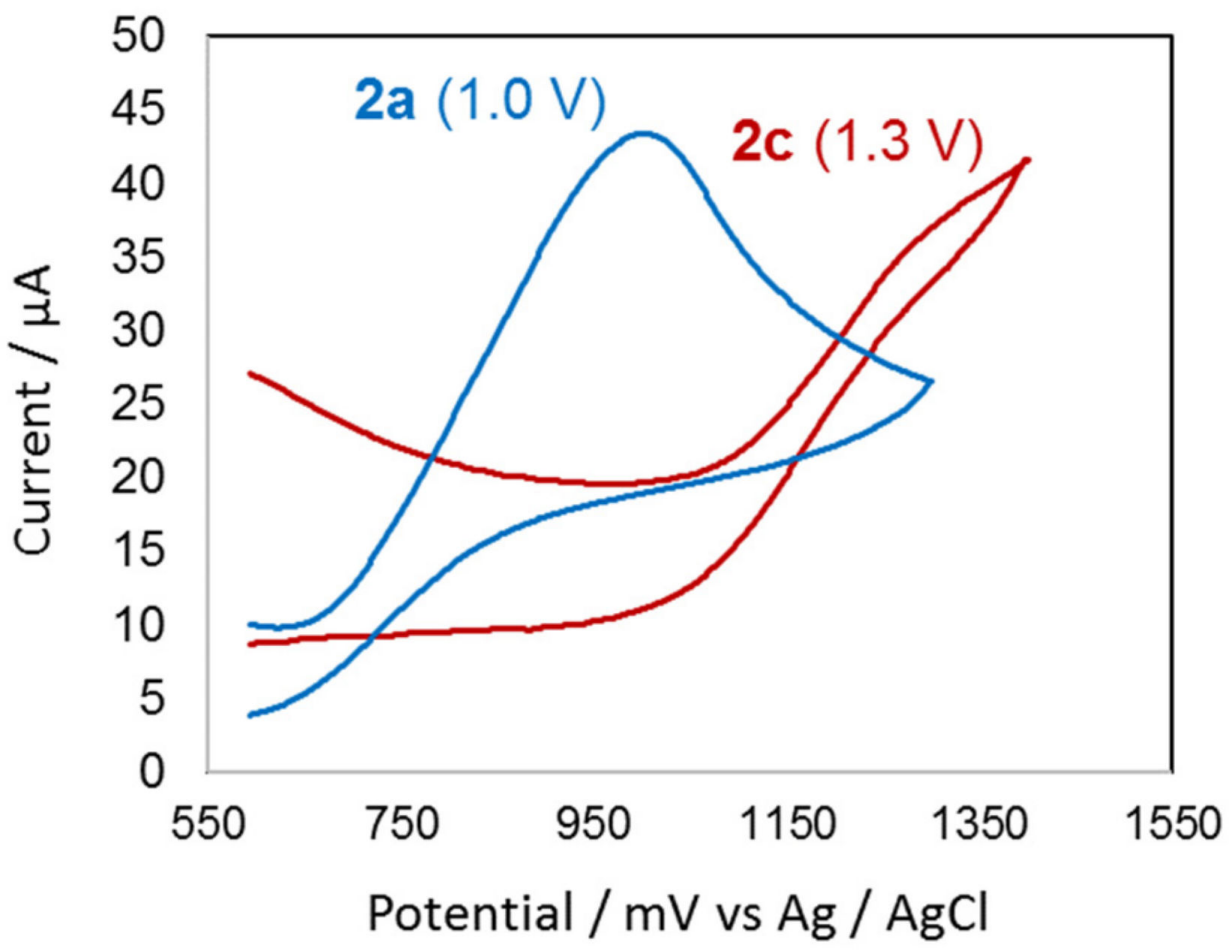
Redox behavior of NiBr(IPr)(PPh_3_) (**2a**) (blue line) and **2c** (red line) in THF with Bu_4_NPF_6_ (working electrode: Pt; counter electrode: Pt; scan rate: 100 mV·s^−1^).

It was found that the stability of **2c** in air was improved more than that of **2a**. In fact, in the solid state, it had not been degraded by oxidation for a period of time: the surface crystal color of **2c** began to change gradually after several minutes upon oxidation in air, whereas **2a** was oxidized as soon as it was exposed to air. On the other hand, the resistance to oxidation could be evaluated by cyclic voltammogram (CV), which was measured with THF solutions of **2a** or **2c**, an excess amount of free phosphine (2 equiv), and Bu_4_NPF_6_. The free phosphine (PPh_3_ for **2a** and P(Ar*_m_*-_CF3_)_3_ for **2c**) was added in order to suppress the generation of the Ni(I) dimer **1** with the liberation of phosphine in equilibrium. As shown in Figure 3, in the CV measured using Ag/AgCl as a reference electrode, the one-electron oxidation potential of **2a** was 1.0 V, and that of **2c** was higher, 1.3 V. These were not oxidation potentials of the free phosphines. Therefore, it is believed that the oxidation resistance of the nickel center is shown to be improved by the fluorine-substituted phosphine, which may also be reflected in the stability in the solid state, along with the fluorine-derived inter- or intramolecular interactions.

### 2.4. Catalytic Performance for Cross-Coupling Reaction

**Scheme 3:**
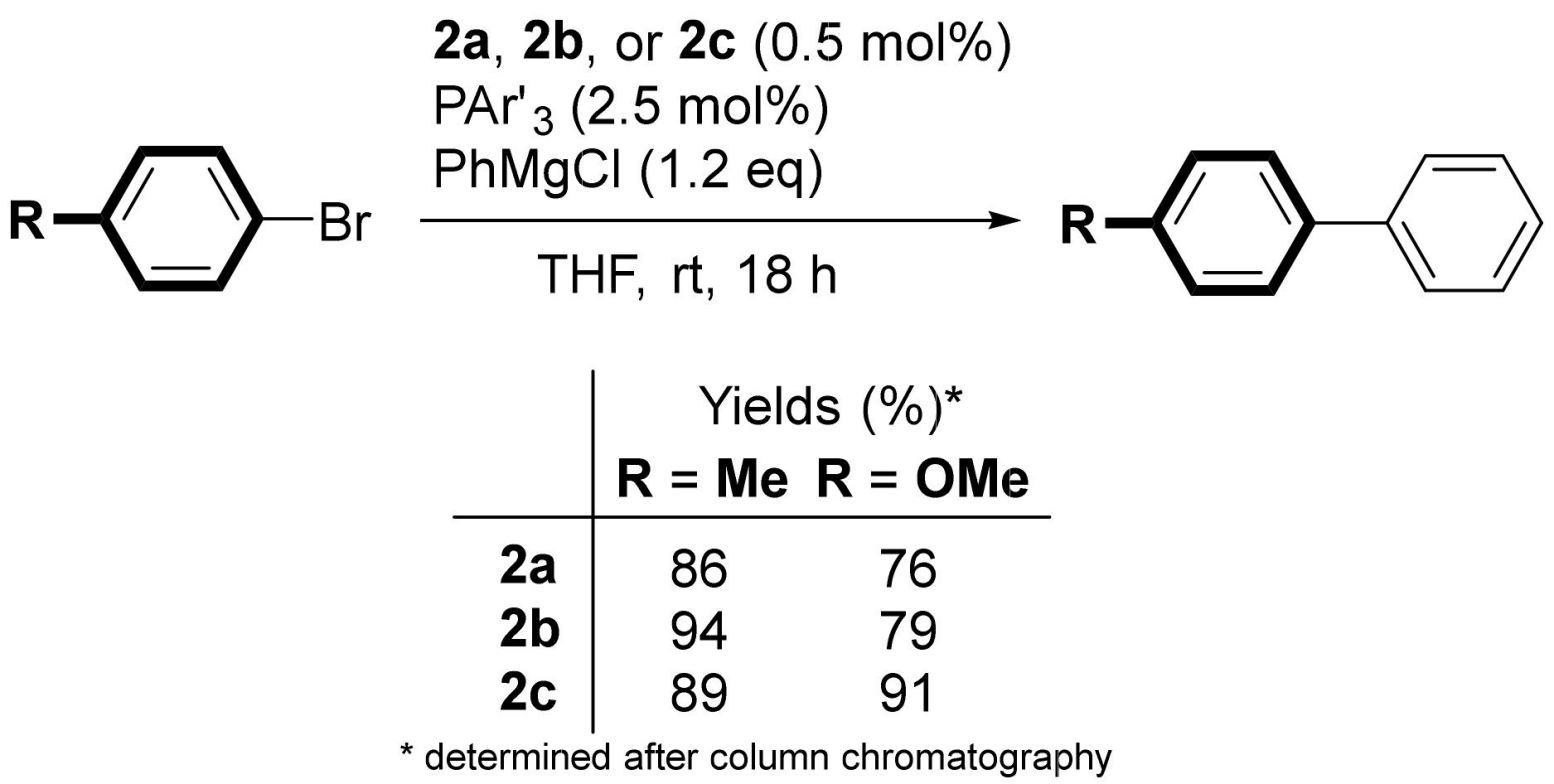
Kumada–Tamao–Corriu coupling of aryl bromides using **2a**–**2c** as catalysts.

The Kumada–Tamao–Corriu coupling of aryl bromides using complexes **2a**, **2b**, and **2c** were representatively conducted under the same reaction conditions. Because the equilibrium ratio of the regenerated dimer **1** observed in the ^1^H-NMR spectrum was only slightly different between **2a** and **2c**, it is expected that the catalytic activity of these complexes will not change. As shown in Scheme 3, 0.5 mol% of **2a**–**2c** were added to the reaction media in the presence of 5 equiv of the corresponding triarylphosphine, and the mixture was stirred under an inert gas atmosphere at room temperature for 18 h. The addition of the excess amount of triarylphosphine can shift the ligand-elimination equilibrium from the dimer **1** toward the mononuclear complexes **2**. In the reaction of 4-bromotoluene with phenylmagnesium bromide, the product 1-methyl-4-phenylbenzene was successfully obtained in excellent yields (86, 94, and 89% using **2a**, **2b**, and **2c**, respectively). More inactive substrate (4-bromoanisole) also produced 1-methoxy-4-phenylbenzene in high yields (76, 79, and 91% with **2a**, **2b**, and **2c**, respectively). These results indicated that the reactions proceeded efficiently under these conditions, regardless of the nature of triarylphosphines, as expected.

## 3. Discussion

As noted above, apparently **2c** is more stable in air than **2a** in the crystalline state. It is believed that the stability in the crystalline state is derived from several factors. One of them is a steric effect. X-ray crystallography demonstrated that the bulky *meta*-CF_3_ substituted phosphine buried the coordination sphere around the nickel center other than those of the NHC and bromine, kinetically blocking the facile access of small molecules such as dioxygen. As is supportde by the discussion above, more hindered, *ortho*-fluorine substituted P(C_6_F_5_)_3_ was difficult to coordinate to nickel. Additionally, the increase in the oxidation potential from Ni(I) to Ni(II) for **2c** should lead to the resistance of the nickel center to oxidation in air, compared with **2a**. However, in solution, the stability of the complex derived from the phosphine should be greatly reduced by its facile elimination to form the Ni(I) dimer **1**, enabling a similar catalyst performance of **2c** to that of **2a** in Kumada–Tamao–Corriu coupling of aryl bromides via the dinickel reaction pathway [9].

**Figure 4:**
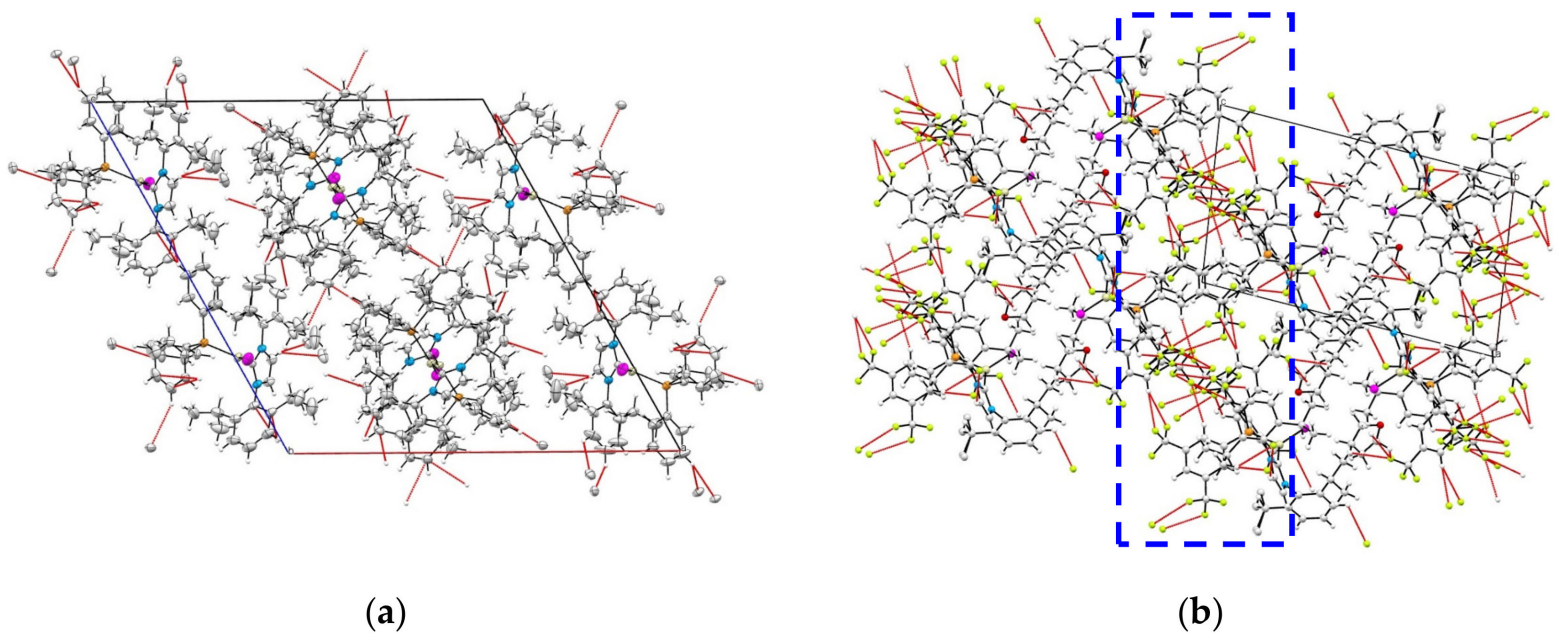
Crystal packing views from the (010) planes depicted using crystallographic data of (**a**) **2a** and (**b**) **2c** (pink: Br; gray: C; light green: F; red: O; orange: P; light yellow: Ni). Short contacts that are shorter than van der Waals radii are addressed as red lines. A blue dashed line in (**b**) highlighted a layer where many contacts containing F atoms (light green) are gathered.

As indicated in the literature, the presence of intermolecular interactions in the crystals formed with fluorine atoms may not be negligible [29]. This hypothesis for the specific intermolecular interactions was strongly supported by the results of X-ray crystallography. It should be noted that there are characteristic, multiple short contacts involving fluorine atoms between **2c** molecules, such as the F···H interaction observed with hydrogens of aryl *para*-C–H in phosphine and isopropyl CH_3_ groups in IPr, as well as many F···F interactions (Figure 4b and ESI). Such F···F interactions have been visualized as a strong dispersed interaction (i.e. van der Waals interaction) in perfluoropolymers [38] and perfluoroalkanes [39]. Interestingly, such short contacts were mostly distributed in layers, suggesting that these interactions could be a driving force to induce and subsequently stabilize the crystal packing. In this packing structure, the CF···π interaction was not observed, in contrast to the palladium chemistry [29]. On the other hand, in a crystal of **2a**, the fewer intermolecular short contacts were observed only between C–H groups (Figure 4a and ESI). It was difficult to evaluate the strength of the intermolecular interactions experimentally, because thermal measurements such as differential scanning calorimetry (DSC) or thermo gravimeter (TG) may break the metal–phosphorus and metal–carbon bonds more easily. However, it is believed that the higher oxidation potential and these steric and electronic factors specifically derived from *meta*-CF_3_ substituted phosphine contribute to the air-stable nature of **2c** in the solid state compared with that of **2a**, at present.

## 4. Materials and Methods

### 4.1. Materials

Super-dehydrated grade THF, toluene, and hexane were used as solvents as purchased from WAKO Pure Chemical Industries, Ltd., Tokyo, Japan. Benzene-*d*_6_ was distilled from sodium benzophenone ketyl and stored under a nitrogen atmosphere. Organic reagents used for coupling reactions were distilled just before use or used as purchased. *N*-Heterocyclic carbene (IPr) [40] and nickel dimer, [Ni(IPr)(μ-Br)]_2_ (**1**) [35], were prepared according to the literature methods.

#### 4.1.1. Ni(IPr)(PPh_3_)Br (**2a**)

Complex **1a** (66.5 mg, 0.0630 mmol) and PPh_3_ (33.0 mg, 0.126 mmol) were dissolved in THF (1 mL) at room temperature, and the mixture was stirred for 5 min to give a light-yellow solution. Hexane (4 mL) was then added to the obtained solution, and the solution was stored at −30 °C. Yellow crystals were obtained in a 42% yield (42.0 mg) after filtration. ^1^H-NMR (C_6_D_6_): *δ* 11.0 (brs), 8.5 (brs), 4.5 (brs), 1.7 (brs). Anal. calcd. for C_45_H_51_N_2_PNiBr: C 68.46%, H 6.51%, N 3.55%; Found: C 67.78%, H 6.47%, N 3.51%.

#### 4.1.2. Ni(IPr)(PAr_(*p*-CF3)3_)Br (**2b**)

Complex **1a** (52.5 mg, 0.0500 mmol) and PAr_(*p*-CF3)3_ (46.9 mg, 0.100 mmol) were dissolved in THF (1 mL) at room temperature, and the mixture was stirred for 5 min to give an orange-red solution. Hexane (4 mL) was then added to the obtained solution, and the solution was stored at −30 °C. Orange crystals were obtained in a 57% yield (63.7 mg) after filtration. ^1^H-NMR (C_6_D_6_): *δ* 11.0 (brs), 8.4 (brs), 4.5 (brs), 1.8 (brs). ^19^F-NMR (C_6_D_6_): *δ* −53.6. Anal. calcd. for C_48_H_48_N_2_F_9_PNiBr: C 58.03%, H 4.87%, N 2.82%; Found: C 58.48%, H 4.48%, N 2.72%.

#### 4.1.3. Ni(IPr)(PAr_(*m*-CF3)3_)Br (**2c**)

Complex **1a** (44.0 mg, 0.418 mmol) and PAr_(*m*-CF3)3_ (62.0 mg, 0.835 mmol) were dissolved in THF (1 mL) at room temperature, and the mixture was stirred for 5 min to give an orange-red solution. Hexane (4 mL) was then added to the obtained solution, and the solution was stored at −30 °C. Orange crystals were obtained in a 57% yield (63.7 mg) after filtration. ^1^H-NMR (C_6_D_6_): *δ* 8.3 (brs), 8.0 (brs), 7.5 (brs), 4.5 (brs), 2.0 (brs). ^19^F-NMR (C_6_D_6_): *δ* −63.1. ^31^P-NMR (C_6_D_6_): No signal. Anal. calcd. for C_51_H_45_N_2_F_18_PNiBr: C 51.15%, H 3.79%, N 2.34%; Found: C 51.61%, H 4.02%, N 2.29%.

### 4.2. Methods

All experiments were carried out under an inert gas atmosphere using standard Schlenk techniques and a glove box (MBraun UniLab, München, Germany) unless otherwise noted. Column chromatography of organic products was carried out using silica gel (Kanto Kagaku, silica gel 60 N (spherical, neutral), Tokyo, Japan). The ^1^H-NMR spectra were taken with a Bruker Avance-III400 Y plus 400 MHz spectrometer (Bruker BioSpin, Billerica, MA, USA) at room temperature. Chemical shifts (δ) were recorded in ppm from the solvent signal. The magnetic properties of the materials were investigated using a Quantum Design MPMS-5S superconducting quantum interference device (SQUID) magnetometer (Quantum Design Inc., San Diego, CA, USA). The elemental analysis was carried out with J-Science CHN Corder JM-11 (J-Science Lab Co. Ltd., Kyoto, Japan), equipped with AUTO-SAMPLER, using tin foil, where the samples were held in a glove box. The X-band EPR measurements were collected with a Bruker EMX Plus spectrometer (Bruker BioSpin, Billerica, MA, USA) equipped with a continuous flow N_2_ cryostat.

#### 4.2.1. Kumada–Tamao–Corriu Coupling of Aryl Bromides

In a typical example, 4-bromotoluene (136.8 μL, 0.80 mmol), triphenylphosphine (5.3 mg, 0.020 mmol), and **2a** (4.1 mg, 2.0 μmol) were dissolved in THF (1 mL). After stirring for 5 min, phenyl magnesium chloride THF solution (0.60 mL, 1.2 mmol) was added to the solution. After 18 h, water (20 mL) was added. The organic layer was extracted with dichloromethane (20 mL × 4). The residual product was purified with silica gel column chromatography eluting with hexane to give 4-methoxybiphenyl (white solid; 115.6 mg, yield 86%).

#### 4.2.2. X-ray Crystallography

Single crystals of **2a**, **2b**, and **2c** for X-ray diffraction were grown at −30 °C from toluene/hexane (**2a**) and THF/hexane (**2b** and **2c**) solutions. All the data were obtained at 125 (**2a**), 151 (**2b**), and 180 K (**2c**) using a Rigaku Saturn CCD diffractometer with a confocal mirror and graphite-monochromated Mo Kα radiation (λ = 0.71070 Å). Data reduction of the measured reflections was performed using the software package CrystalStructure [41]. The structures were solved by direct methods (SHELXT-2014) [42] and refined by full-matrix least-squares fitting based on F^2^, using the program SHELXL-2014 [43]. All non-hydrogen atoms were refined with anisotropic displacement parameters. All hydrogen atoms were located at ideal positions and included in the refinement but were restricted to riding on the atom to which they were bonded. Unfortunately, the refinement for the crystal **2b** cannot be completed, and only the preliminary structure was shown in the supporting information. CCDC 1947417–1947418 contains the supplementary crystallographic data of **2a** and **2c** for this paper. A copy of the data can be obtained free of charge via http://www.ccdc.cam.ac.uk/cgi-bin/catreq.cgi.

#### 4.2.3. Theoretical Details

All the DFT calculations were performed utilizing the GAUSSIAN 16 Rev. A.03 program package (Gaussian Inc., Wallingford, CT, USA) [37]. The B3LYP functional was employed with a standard split valence-type basis set, 6-31G(d,p). The single-point calculations to obtain SOMOs were carried out using crystallographic coordinates without geometry optimization, and done with a tight self-consistent field (SCF) convergence criterion. All the computation was carried out using the computer facilities at Research Institute for Information Technology, Kyushu University, Fukuoka, Japan.

#### 4.2.4. Electrochemistry

The cyclic voltammogram of **2a** and **2c** were recorded on an ALS/chi electrochemical analyzer Model/610A with a platinum working electrode, a silver wire reference electrode, and a platinum wire counter electrode, with a scan rate of 50 mV·s^−1^. The analyte solutions of these complexes were prepared with a 0.1 M solution of tetra-*n*-butyl ammonium perchlorate in acetonitrile. Ferrocene was used as an internal standard and the potential reported here is referenced to the ferrocene/ferricinium couple.

## 5. Conclusions

In summary, the Ni(I)-IPr complex bearing *meta*-CF_3_ substituted triarylphosphine ligand was successfully isolated. Although the “superstable” Pd(0) catalyst has been derived using palladium and phosphine, such a triarylphosphine complex using nickel has been determined here for the first time. In the solid state, steric bulk, appropriate electron-withdrawing properties, and the presence of intermolecular interactions make nickel less susceptible to oxidation in air where the triarylphosphine is coordinated. Particularly, intermolecular multiple interactions including those between F and H, and F and F were found to be gathered in layers in the crystalline state. On the other hand, it is possible to maintain the same catalytic activity as the analogous PPh_3_ complex, since the phosphine can be easily eliminated in solution, resulting in the generation of active species. In the future, we plan to work on the development of other useful catalytic reactions and detailed mechanistic research using this Ni(I) complex.

## Figures and Tables

**Table 1:** Representative bond lengths and angles of **2a** and **2c**.

		2a	2c
Lengths (Å)	Ni(1)–Br(1)	2.301(1)	2.3016(8)
	Ni(1)–P(1)	2.201(1)	2.182(1)
	Ni(1)–C(1)	1.918(5)	1.936(3)
Angles (°)	C(1)–Ni(1)–Br(1)	138.4(1)	137.1(1)
	C(1)–Ni(1)–P(1)	114.9(1)	113.5(1)
	Br(1)–Ni(1)–P(1)	106.44(4)	109.29(4)

## Supplementary Materials

The supplementary materials are available online. Figure S1–S8: NMR spectra for the complexes, Figure S9: χ_mol_*T* vs *T* plot, Figure S10: ESR spectrum, Figure S11–S12: Packing views of the complexes (expanded views), details of the Kumada coupling reactions including NMR spectra of products.
