# Supplementary material for: Fluorine-Substituted Arylphosphine for an NHC-Ni(I) System, Air-Stable in a Solid State but Catalytically Active in Solution
Source: Molecules. 2019 Sep 4;24(18):3222. doi: 10.3390/molecules24183222 (PMC6766797; doi:10.3390/molecules24183222)
Supplement: Supplementary file 1 [file molecules-24-03222-s001.pdf]

molecules

Supplementary Materials

Fluorine-Substituted Arylphosphine for NHC-Ni(I) System, Air-Stable in Solid State but Catalytically Active in Solution

Kouki Matsubara [1,*], Takahiro Fujii [1], Rion Hosokawa [1], Takahiro Inatomi [1], Yuji Yamada [1] and Yuji Koga [1]

[1] Department of Chemistry, Fukuoka University, 8-19-1 Nanakuma, Fukuoka 814-0180, Japan; ygyamada@fukuoka-u.ac.jp (Y.Y.); y-koga@fukuoka-u.ac.jp (Y.K.)

* Correspondence: kmatsuba@fukuoka-u.ac.jp; Tel.: +81-92-871-6631

Contents:

S-1. NMR Spectra of Ni(I) Complexes.

S-2. SQUID and EPR Spectra for **2c**.

S-3. Preliminary X-ray Crystal Structure for **2b**.

S-4. Packing Views Containing Short Contacts of 2a and **2c** (Expanded Views).

S-5. Kumada-Tamao-Corriu Coupling of Aryl Bromides.

S-1. NMR Spectra of Ni(I) Complexes

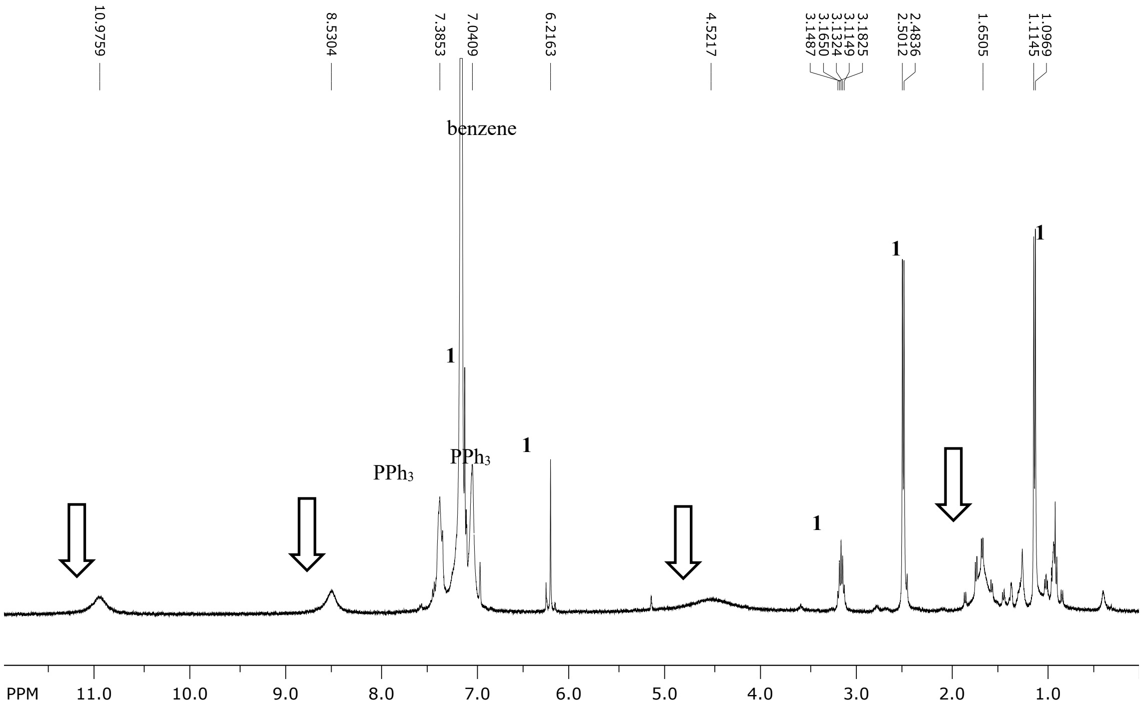

Figure S1. ^{1}H-NMR spectrum (400 MHz, C_6D_6, 298 K) for crystals of **2a** (arrows point to the signals). Complex **1** and phosphine were generated in situ as a result of equilibrium.

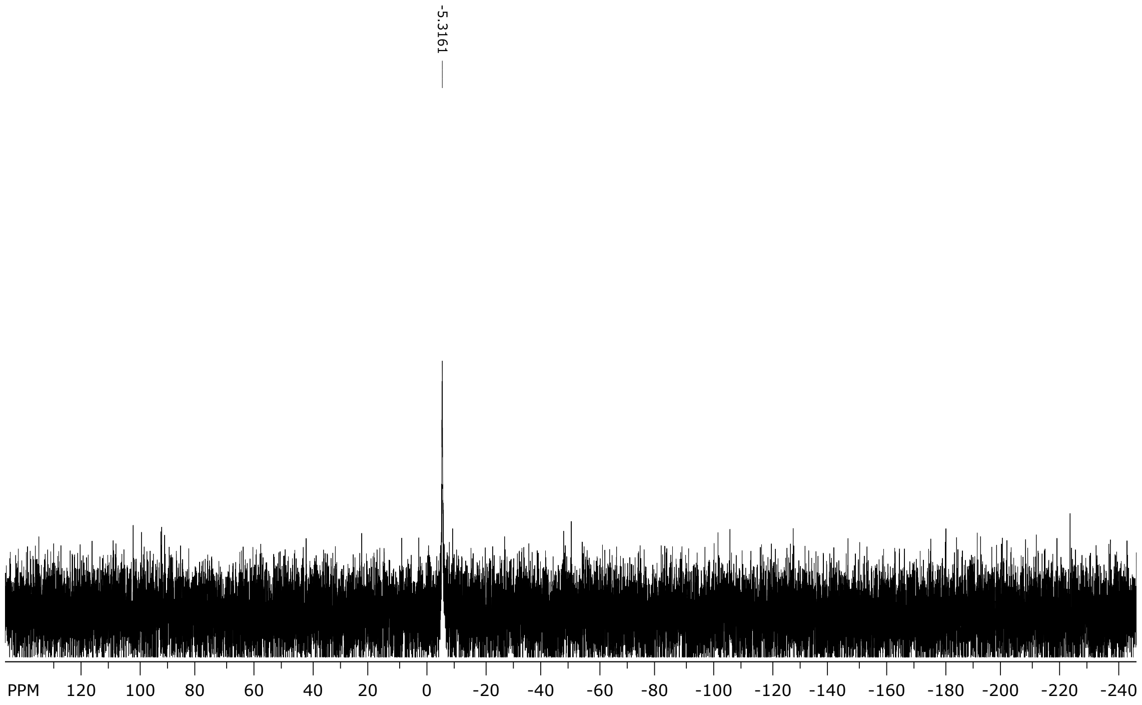

Figure S2. ^{31}P-NMR spectrum (162 MHz, C_6D_6, 298 K) for crystals of **2a**. A signal from free phosphine at δ -6.0 was observed as a result of equilibrium.

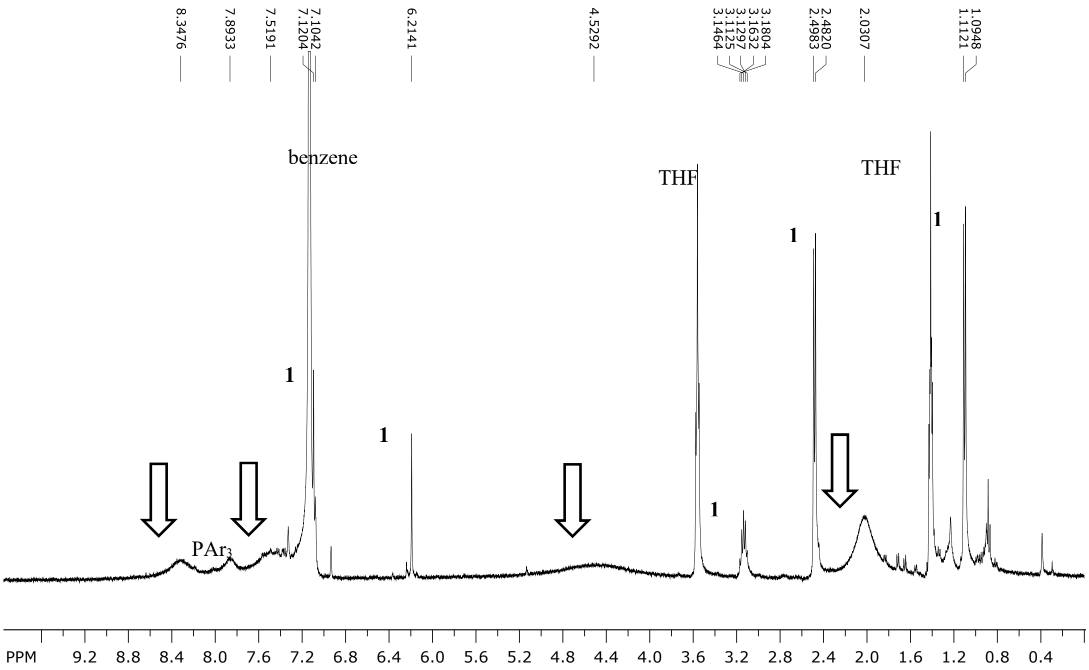

Figure S3. ^{1}H-NMR spectrum (400 MHz, C_6D_6, 298 K) for crystals of **2b** (arrows point to the signals). Complex **1** and phosphine were generated in situ as a result of equilibrium. THF molecule was contaminated in the crystals because of co-crystallization in THF solution.

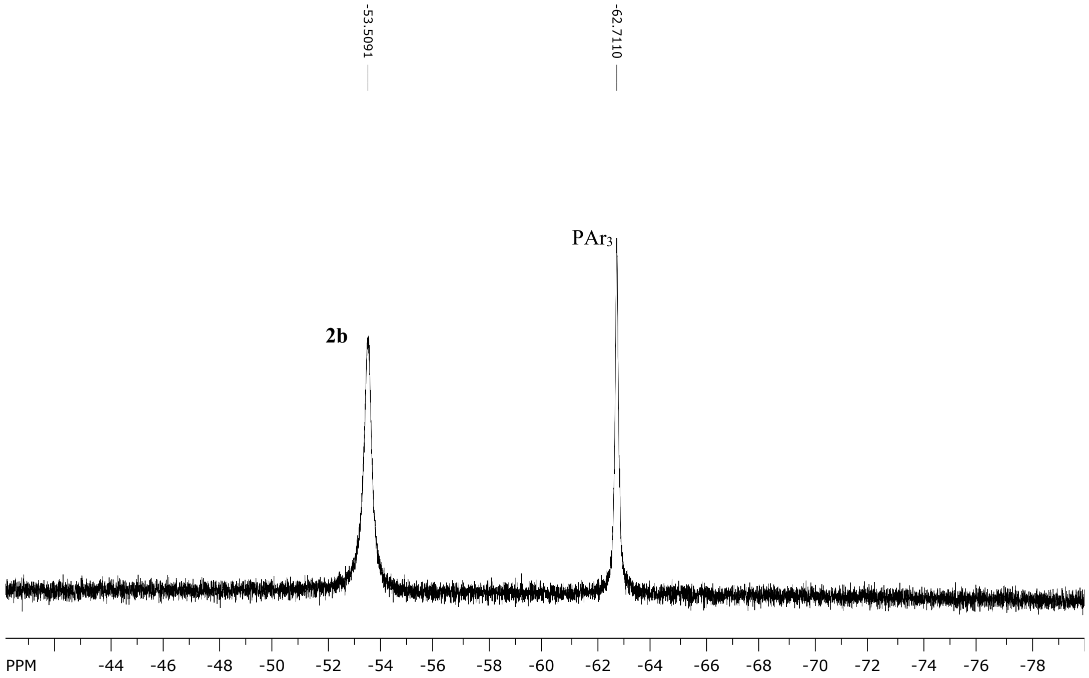

Figure S4. ^{19}F-NMR spectrum (376 MHz, C_6D_6, 298 K) for crystals of **2b**.

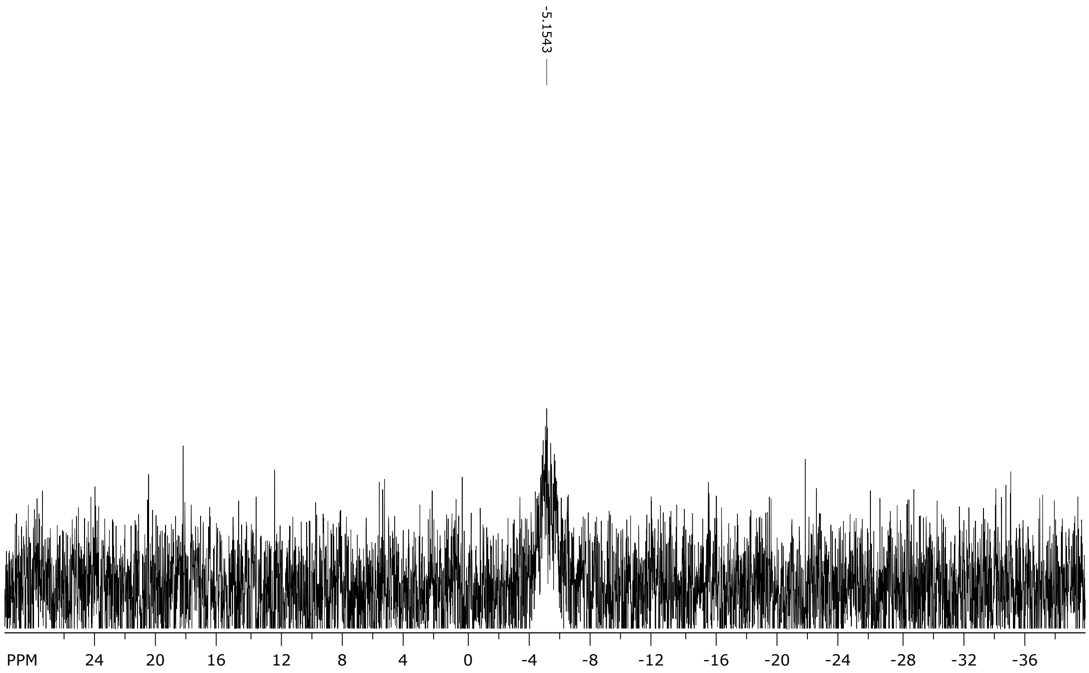

Figure S5. ^{31}P-NMR spectrum (162 MHz, C_6D_6, 298 K) for crystals of **2b**. A signal from free phosphine at δ -6 was observed as a result of equilibrium.

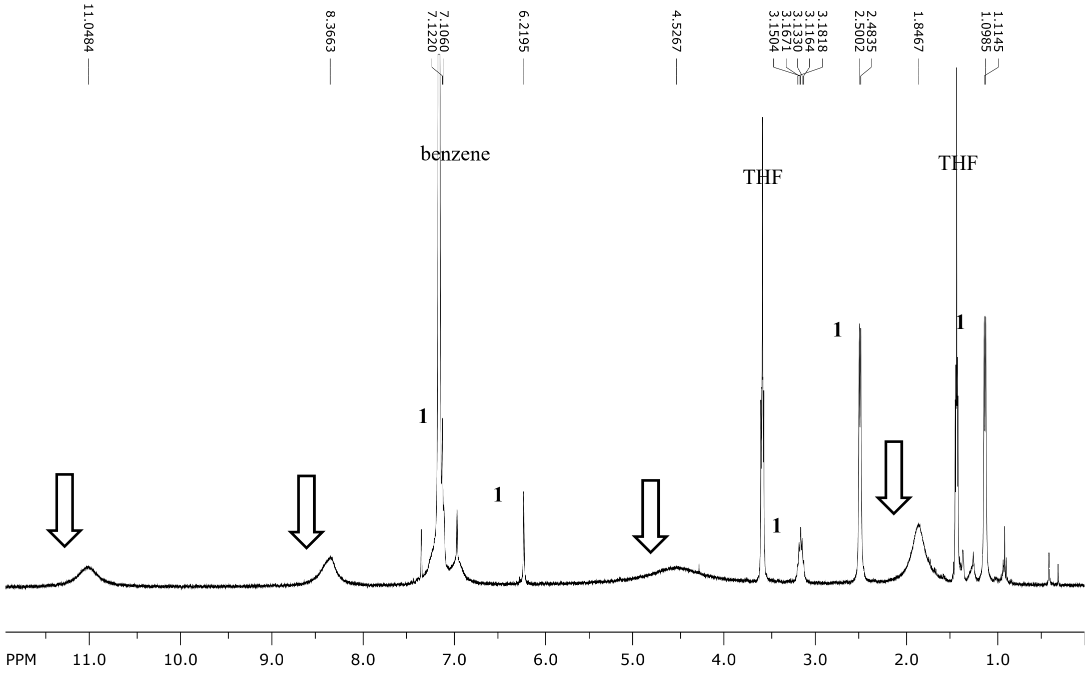

Figure S6. ^{1}H-NMR spectrum (400 MHz, C_6D_6, 298 K) for crystals of **2c** (arrows point to the signals). Complex **1** and phosphine were generated in situ as a result of equilibrium. THF molecule was contaminated in the crystals because of co-crystallization in THF solution.

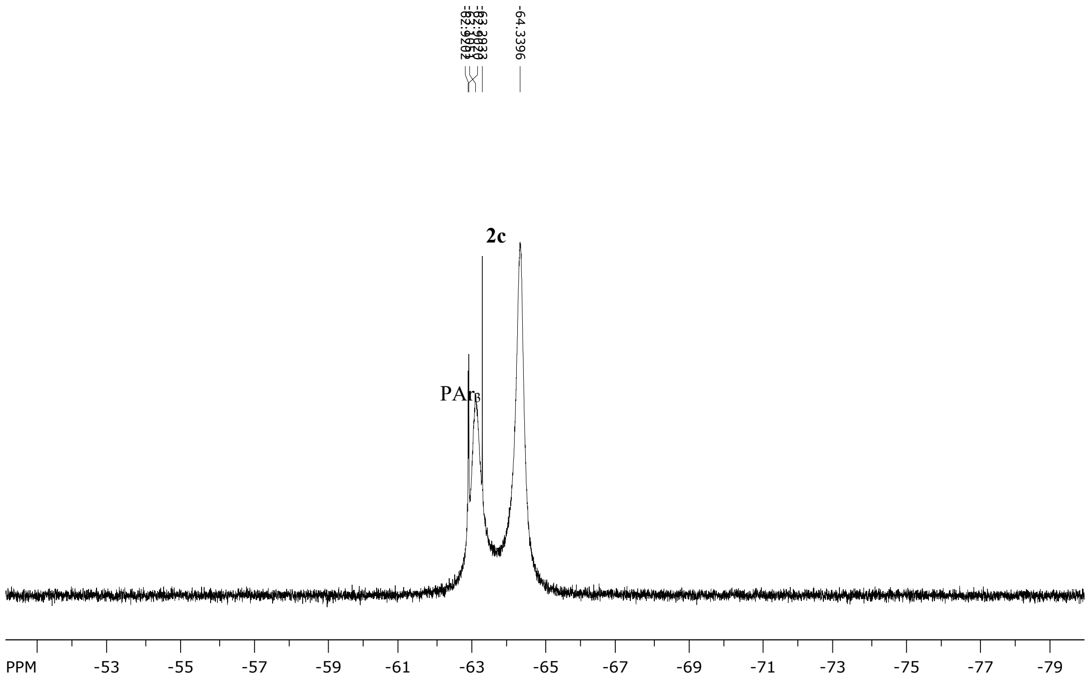

Figure S7. ^{19}F-NMR spectrum (376 MHz, C_6D_6, 298 K) for crystals of **2c**. A signal from free phosphine was broadened probably because of slow equilibrium in the timescale of aquisition.

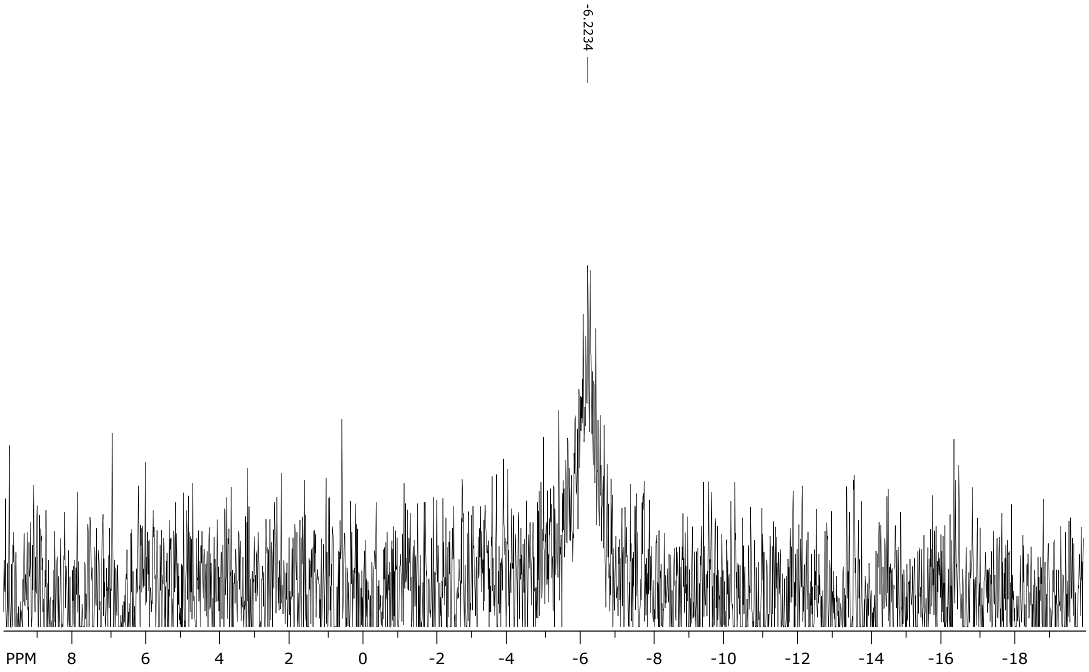

Figure S8. ^{31}P-NMR spectrum (162 MHz, C_6D_6, 298 K) for crystals of 2c. A signal from free phosphine at δ -6 was observed as a result of equilibrium.

S-2. SQUID and EPR spectra for 2c

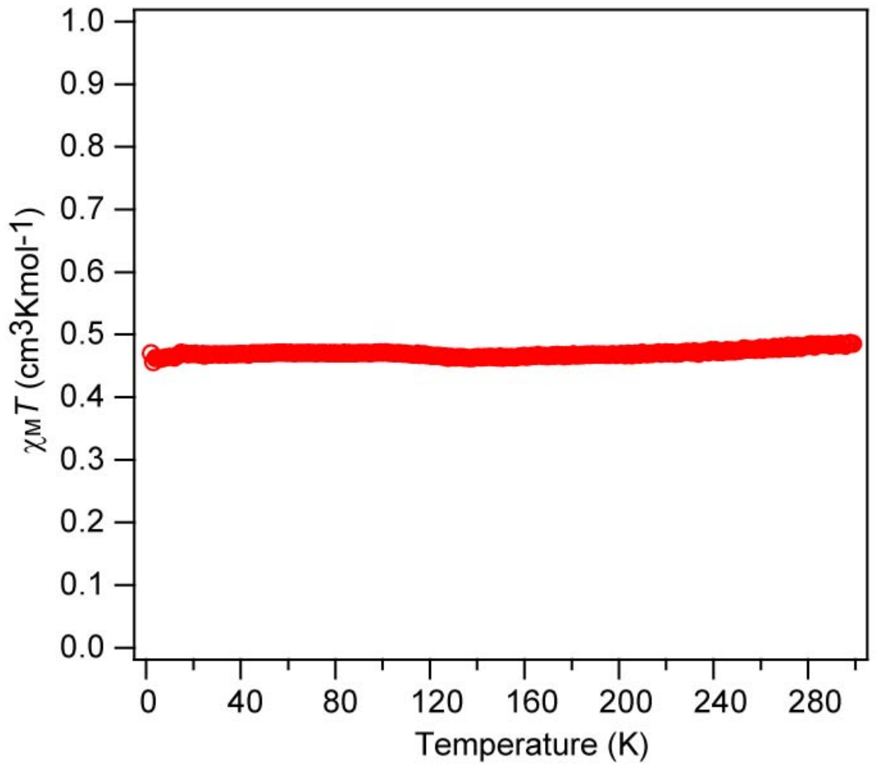

Figure S9. $\chi_{mol}T$ vs. T plot for **2c**, obtained from SQUID measurement ($\chi_{mol}T$ = 0.46-0.48).

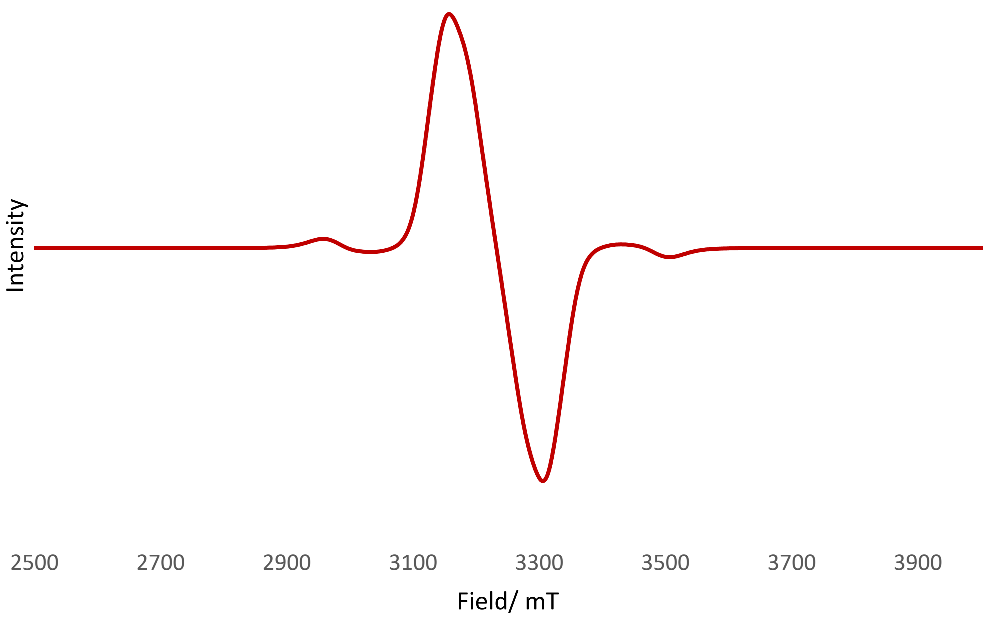

Figure S10. ESR Spectrum for bulk **2c** at 90 K (−183 °C) (g_{xx} = 2.251, g_{yy} = 2.087, g_{zz} = 1.943).

S-3. Preliminary X-ray Crystal Structure for 2b

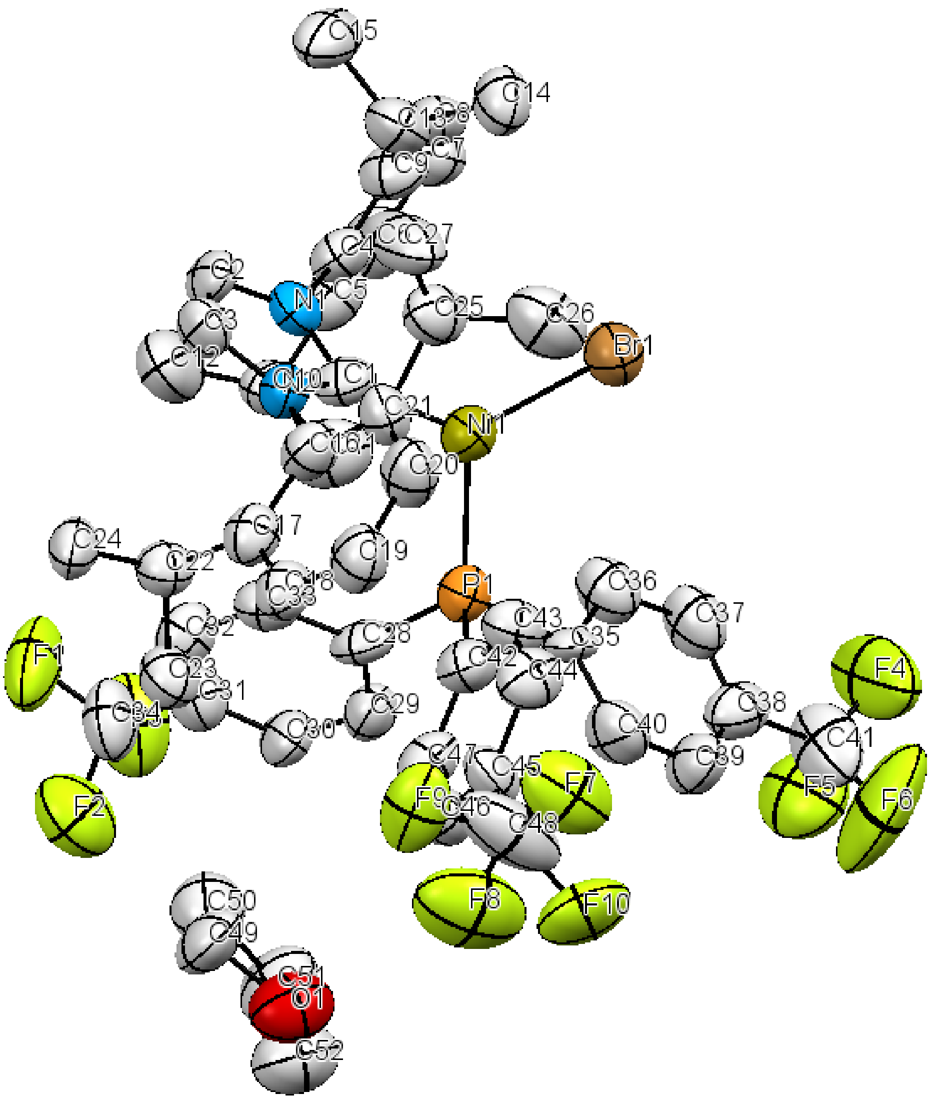

Figure S11. ORTEP drawing of **2b**, as a preliminary refined structure (50% thermal ellipsoids, Space group: $P2_1/n$, R1 = 0.2052, wR2 = 0.5530). Hydrogen atoms are omitted for clarity.

S-4. Packing Views Containing Short Contacts of 2a and 2b

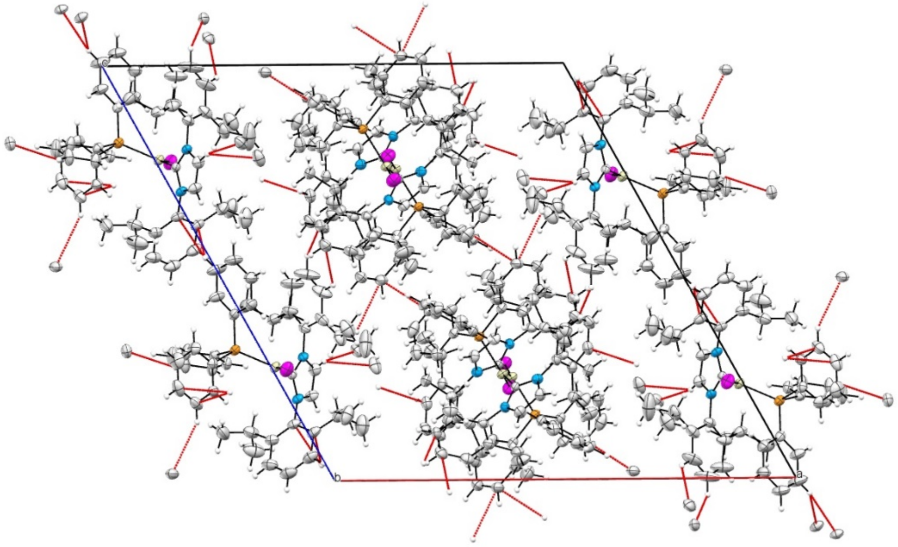

Figure S12. Packing view from the a-c plane using a softwere Mercury Ver 3.9 (for Windows by CCDC) depicted with crystallographic data of **2a**. Short contacts which are shorter than van der Waals radii are addressed as red bonds.

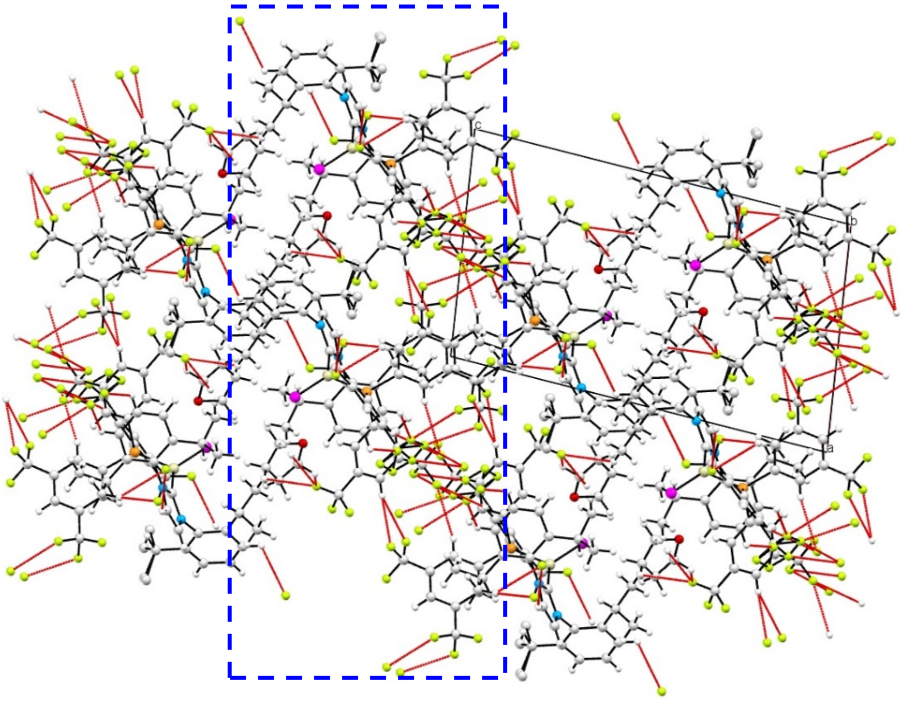

Figure S13. Packing view from the a-c plane using a softwere Mercury Ver 3.9 (for Windows by CCDC) depicted with crystallographic data of **2c**. Short contacts which are shorter than van der Waals radii are addressed as red bonds. A blue square highlighted a layer where many contacts containing F atoms (light green) are gathered.

S-5. Kumada-Tamao-Corriu Coupling of Aryl Bromides

Reaction of 4-bromotoluene using 2a

In a typical example, 4-bromotoluene (136.8 μL, 0.80 mmol), triphenylphosphine (5.3 mg, 0.020 mmol), and **2a** (4.1 mg, 4.0 μmol) were dissolved in THF (1 mL). After stirring for 5 min, phenyl magnesium chloride THF solution (0.60 mL, 1.2 mmol) was added to the solution. After 18 h, water (20 mL) was added. The organic layer was extracted with dichloromethane (20 mL × 4). The residual product was purified with silica gel column chromatography eluting with hexane to give 1-methyl-4-phenylbenzene (white solid; 115.6 mg, 86%).

^{1}H-NMR (400 MHz, $CDCl_3$, 298 K): δ = 7.53–7.55 (m, 2 H), 7.44–7.46 (m, 2 H), 7.36–7.40 (m, 2 H), 7.26–7.30 (m, 1 H), 7.19–7.22 (m, 2 H), 2.35 (s, 3 H) ppm. ^{13}C-NMR (101 MHz, $CDCl_3$, 298 K): δ = 141.3, 138.5, 137.2, 129.6, 128.9, 127.1, 127.1, 21.2 ppm.

Reaction of 4-bromotoluene using 2b

4-Bromotoluene (136.8 μL, 0.800 mmol), tris(4-(trifluoromethyl)phenyl)phosphine (9.3 mg, 0.020 mmol), **2b** (4.5 mg, 4.0 μmol), and phenyl magnesium chloride THF solution (0.60 mL, 1.2 mmol) were used: 126.6 mg, 94% yield.

Reaction of 4-bromotoluene using 2c

4-Bromotoluene (136.8 μL, 0.800 mmol), tris(3,5-bis(trifluoromethyl)phenyl)phosphine (13.4 mg, 0.020 mmol), **2c** (4.7 mg, 4.0 μmol), and phenyl magnesium chloride THF solution (0.60 mL, 1.2 mmol) were used: 119.5 mg, 89%.

Reaction of 4-bromoanisole using 2a

In a typical example, 4-bromoanisole (100 μL, 0.80 mmol), triphenylphosphine (5.2 mg, 0.020 mmol), and **2a** (3.0 mg, 4.0 μmol) were dissolved in THF (1 mL). After stirring for 5 min, phenyl magnesium chloride THF solution (0.60 mL, 1.2 mmol) was added to the solution. After 18 h, water (20 mL) was added. The organic layer was extracted with dichloromethane (20 mL x 4). The residual product was purified with silica gel column chromatography eluting with hexane to give 1-methoxy-4-phenylbenzene (white solid; 117.0 mg, 79% yield).

^{1}H-NMR (400 MHz, $CDCl_3$, 298 K): δ = 7.52–7.56 (m, 4 H), 7.39–7.43 (m, 2 H), 7.28–7.32 (m, 1 H), 6.97–6.99 (m, 2 H), 3.86 (s, 3 H) ppm. ^{13}C-NMR (101 MHz, $CDCl_3$, 298 K): δ = 159.3, 141.0, 133.9, 128.8, 128.3, 126.9, 126.8, 114.3, 55.5 ppm.

Reaction of 4-bromoanisole using 2b

4-Bromoanisole (100 μL, 0.80 mmol), tris(4-(trifluoromethyl)phenyl)phosphine (9.3 mg, 0.020 mmol), **2b** (4.1 mg, 4.0 μmol), and phenyl magnesium chloride THF solution (0.60 mL, 1.2 mmol) were used: 112.6 mg, 76% yield.

Reaction of 4-bromoanisole using 2c

4-Bromoanisole (100 μL, 0.80 mmol), tris(3,5-bis(trifluoromethyl)phenyl)phosphine (13.4 mg, 0.020 mmol), **2c** (4.7 mg, 4.0 μmol), and phenyl magnesium chloride THF solution (0.60 mL, 1.2 mmol) were used: 133.4 mg, 91% yield.

NMR spectra of coupling products:

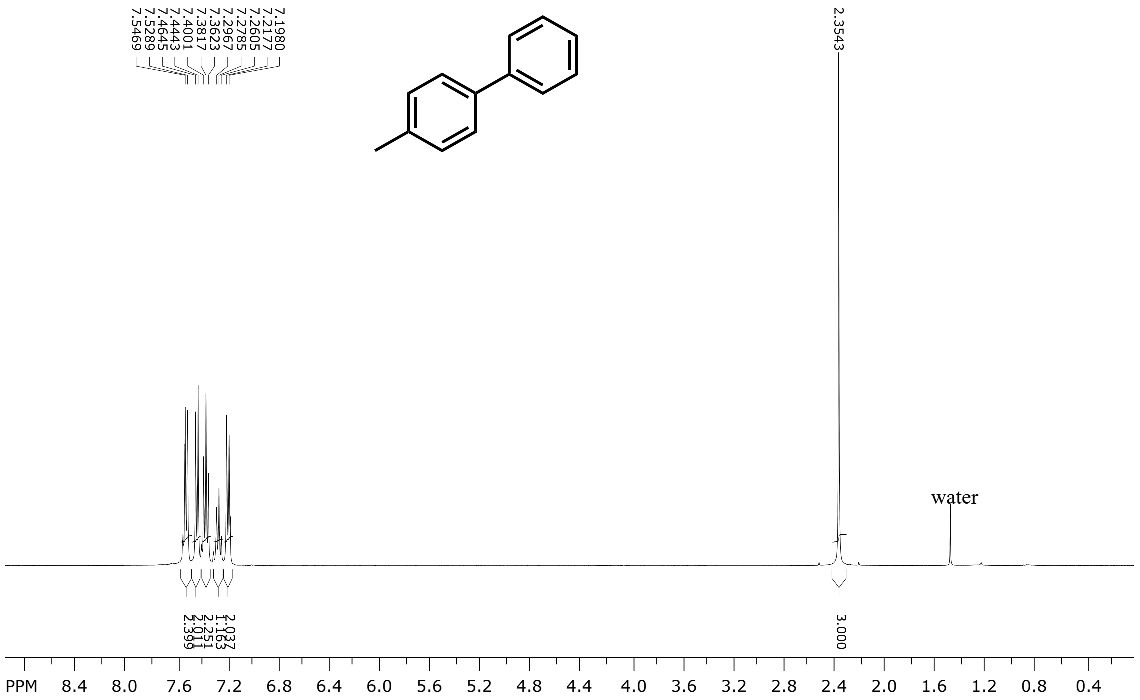

Figure S14. ^{1}H-NMR spectrum for 1-methyl-4-phenylbenzene (400 MHz, $CDCl_3$).

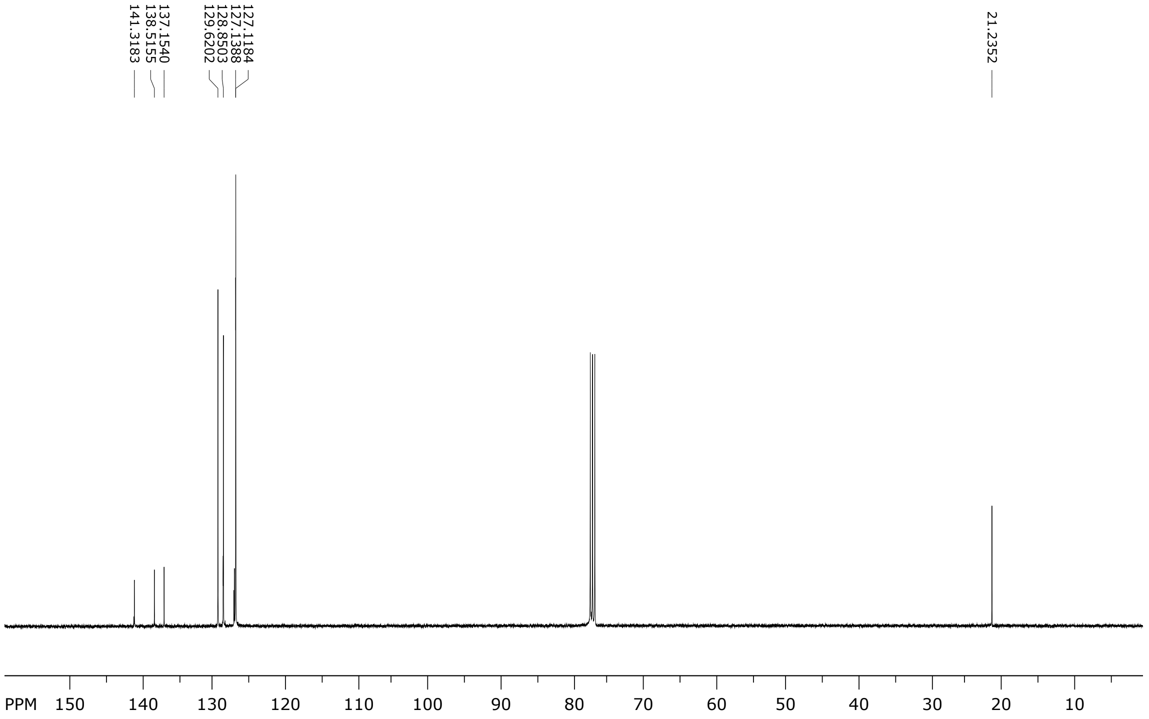

Figure S15. ^{13}C-NMR spectrum for 1-methyl-4-phenylbenzene (101 MHz, $CDCl_3$).

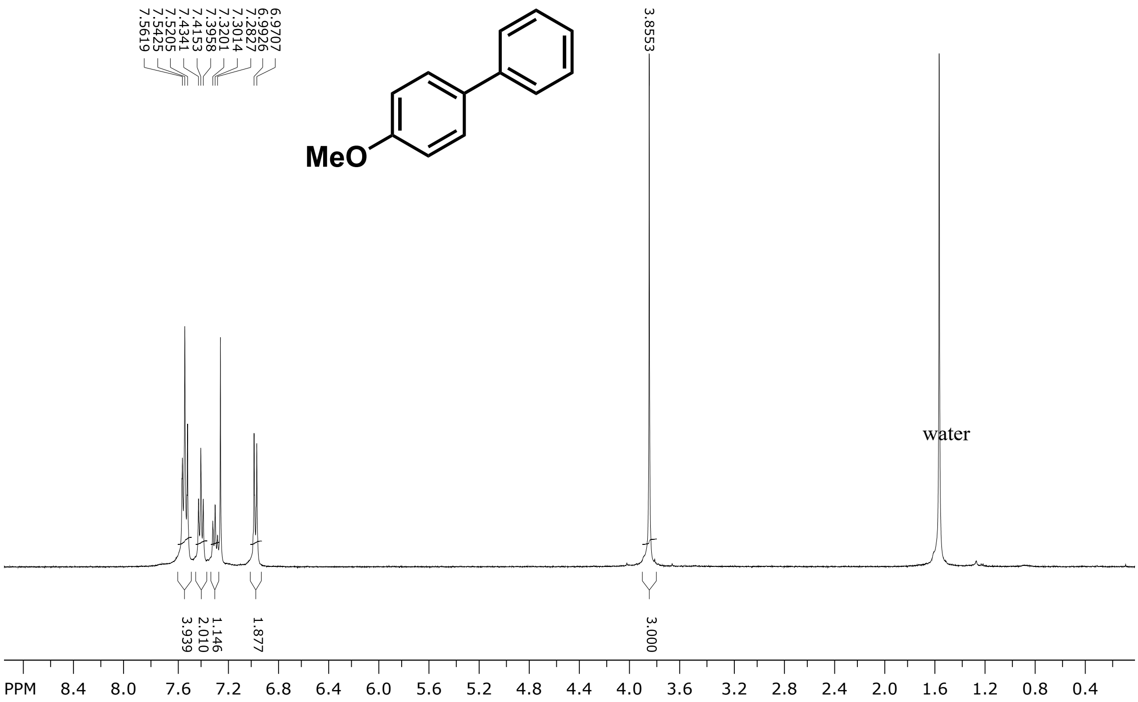

Figure S16. ^{1}H-NMR spectrum for 1-methoxyl-4-phenylbenzene (400 MHz, $CDCl_3$).

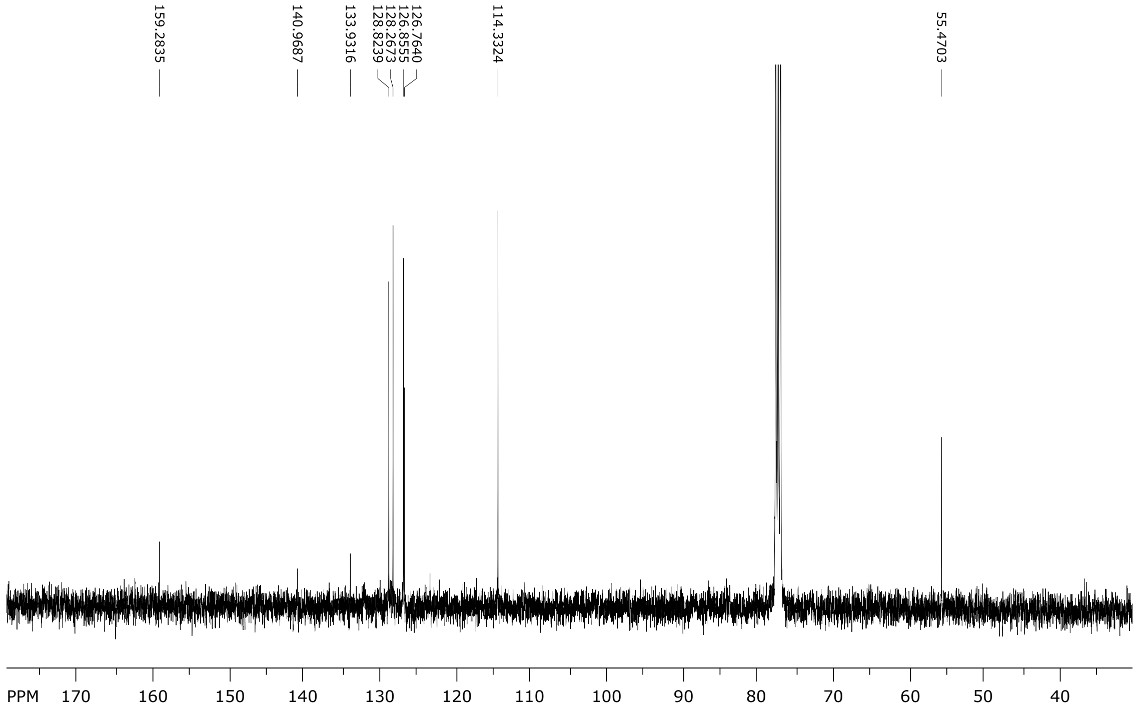

Figure S17. ^{13}C-NMR spectrum for 1-methoxyl-4-phenylbenzene (101 MHz, $CDCl_3$).
